# Circadian Rhythms, Sleep, and Aging: New Visions on the Clock, Sleep, and Aging

**DOI:** 10.1093/geroni/igaa057.2646

**Published:** 2020-12-16

**Authors:** Amita Sehgal

## Abstract

The symposium will highlight the reciprocal relationship between aging and sleep and circadian rhythms. Circadian rhythms and sleep deteriorate with age, but at the same time, disruptions in these processes can contribute too aging. Presenters will show how work in model organisms is providing insight into this relationship

